# The Short-Term Metabolic Modulation of Basil (*Ocimum basilicum* L. cv. ‘Genovese’) after Exposure to Cold or Heat

**DOI:** 10.3390/plants10030590

**Published:** 2021-03-21

**Authors:** Dragana Jakovljević, Jovana Momčilović, Biljana Bojović, Milan Stanković

**Affiliations:** Department of Biology and Ecology, Faculty of Science, University of Kragujevac, 34 000 Kragujevac, Serbia; 1067-2017@pmf.kg.ac.rs (J.M.); biljana.bojovic@pmf.kg.ac.rs (B.B.); mstankovic@kg.ac.rs (M.S.)

**Keywords:** basil, temperature, stress, antioxidant enzymes, secondary metabolites

## Abstract

Chilling stress in spring and mid-season heat stress are important environmental stresses that can significantly affect plant productivity. The objectives of this study were to understand the effects of cold (4 and 10 °C) or heat (30 and 40 °C) stress on biochemical and physiological traits in leaves and roots of basil (*Ocimum basilicum* L. cv. ‘Genovese’) young plants. After short-time exposure to mild and severe temperature stresses, both photosynthetic pigments’ and protein, as well as enzymatic and non-enzymatic defense components in basil leaves and roots, were quantified and compared with the control non-stressed plants. It was shown that both cold and heat treatment increase the content of chlorophyll *a*, chlorophyll *b*, and carotenoids. Chilling correlated with higher content of soluble proteins in leaves, whereas the concentration of these osmoprotectants in roots was higher under both cold and heat stress. For all tested antioxidant enzymes, higher activity was measured in leaves, and activity was related to temperature stress. SOD, CAT, A-POX, and P-POX activities was induced under heat stress, while the higher activity of SOD, CAT, P-POX, and G-POX was recorded under cold stress, compared to the control. In addition to the induced activity of enzymatic components, the content of secondary metabolites including total phenolics, flavonoids, and total anthocyanins, was several times higher compared to the non-stressed plants. Furthermore, total phenolic content was higher in roots than in leaves. Significant positive correlation can be seen among photosynthetic pigments, SOD, total phenolics, and flavonoids under severe temperature stress (4 or 40 °C) in basil leaves, while for roots, positive correlation was found in the content of secondary metabolites and activity of CAT or peroxidases. Obtained results are discussed in terms of phenotyping of *O. basilicum* cv. ‘Genovese’ response to heat and chilling stress, which should contribute to a better understanding of merged responses to cold and heat tolerance of this valuable crop.

## 1. Introduction

Harsh environmental conditions (drought, heat, cold, and nutrient deficiency) are referred to as abiotic stresses and are directly related to plant growth, development, and productivity [1]. In the field, plants are exposed to varying temperatures during the season, among days, while in continental environments with large temperature amplitude, plants may undergo both cold and heat stress during a single day [2]. Since temperature stress affects plants at the cellular, organs, or whole plant levels, as a result, plants have evolved a variety of stress responses and signaling pathways to sense mild changes in ambient temperature and adjust metabolism to prevent damage [2,3].

Heat excess (at least 5 °C above plant optimal growing conditions) affects both leaf tissue and root conductance and exhibits physiological disruption and biochemical changes in the organization of cellular structures and membrane function, as well as changes in the pigment ratio and light-dependent chemical reactions in thylakoids, and it is accompanied by the production of antioxidants and other protective molecules [4,5]. Plant responses to high temperature vary with the degree and duration of stress, as well as with the plant type, and the major tolerance mechanisms that are activated are related to ion transporters, proteins, osmoprotectants, and antioxidants [6]. In plants, disruption of integrity and permeability of the cell membrane is one of the primary destructive effects of cold stress [7]. Cold stress directly affects the photosynthetic machinery through a disbalance between received light photon and their efficiency, causing excessive generation of reactive oxygen species (ROS) which oxidates membrane lipids and damages cell structures [7,8]. Although plant responses to low and high temperatures are often considered antagonistic (acclimation to low temperature is associated with increases in membrane fluidity, whereas increases in heat stress resistance are associated with increases in membrane rigidity), there are several points of merging responses to heat and cold stresses, including an increase of osmolyte concentrations, and accumulation of antioxidants [2].

The overproduction of reactive oxygen species (ROS) is a direct result in stress-induced cellular changes. ROS produced during temperature-stress conditions (superoxide O_2_^−^, hydroxyl radical OH^−^, alkoxyl radicals, hydrogen-peroxide H_2_O_2_, and singlet oxygen ^1^O_2_) are highly reactive and cause damage to proteins, lipids, and carbohydrates, which results in oxidative stress [9]. Excessive generation of ROS is controlled by the antioxidant system including both enzymatic and non-enzymatic components which are also information-rich redox buffers and important signaling components that interact with biomembrane related compartments [9]. The enzymatic antioxidant system in plants includes various enzymes (superoxide dismutase, catalase, peroxidases, etc.), among which activity of superoxide dismutase in dismutation of superoxide anions, together with the activity of catalase and peroxidases in hydrogen peroxide removal, is considered as the first line of antioxidant defense [10]. With several thousand compounds, secondary metabolites from the phenolic group are important non-enzymatic antioxidants that act both in plants, as well as in the human diet [11]. In general, higher activity of antioxidant enzymes and increment of secondary metabolites are among the most important factors included in stress tolerance [1].

Plant species that taxonomically belong to the genus *Ocimum* L. (basil) are among the most economically significant representatives of the Lamiaceae family. The cultivation of therapeutically important species of the *Ocimum* genus has been intensified globally, since they are well known sources of natural products with wide areas of nutritional, medicinal, and pharmaceutical application. Among numerous *Ocimum* species and cultivars, *Ocimum basilicum* L. cv. ‘Genovese’ stands out due to its high content of secondary metabolites, in particular rosmarinic and caffeic acids, a wide range of biological activities, and extensive applications in the food industry [12]. Because of their great adaptability to different types of substrates, basil plants are cultivated worldwide; however, the majority of production is limited to tropical and subtropical climates, since basil originates from tropical and warm areas and grows best in warm Mediterranean conditions and commercial indoor farming with temperatures above 20 °C [13]. It is known that, because of a poor tolerance of young leaves and buds to low temperature in cultivated plants, a sudden frost in early spring causes cold stress and negatively affect the commercial yield, whereas the combination of warmer maximum temperatures leads to earlier budburst and greater risk of damage [8]. Therefore, in order to develop improved crops with complete yield potential, it is important to understand the numerous plant mechanisms and their interactions during abiotic stresses and to explore the ways to improve the temperature tolerance of basil plants [14].

The objective of this study was to evaluate physiological and biochemical responses of *O. basilicum* cv. ‘Genovese’ young plants against cold or heat stress. We hypothesized that the activity of antioxidant enzymes is enhanced both by cold and heat stress and that the level of activity is related to the severity of temperature stress. It was also evaluated whether is temperature stress quantitatively related to the production of phenolics and flavonoids, which would support the assumption of the ubiquitous role of the antioxidant system against temperature stress. The results of this study should contribute to a better understanding of temperature tolerance in basil and provide background information for future cultivation management.

## 2. Results

### 2.1. Content of Photosynthetic Pigments and Soluble Proteins

Under low-temperature stress conditions, an increased concentration of photosynthetic pigments could be seen (Table 1), and a statistically significant increase in the content of chlorophyll a, chlorophyll b, as well as total chlorophyll was noticed under chilling stress (4 °C) comparing to the control. Heat stress also caused an increase in the concentration of photosynthetic pigments with statistical differences, compared to the control. Comparing cold and heat stress treatments, a stronger influence of heat on photosynthetic pigment concentration could be seen than cold, since the highest concentration of pigments was measured under 30 and 40 °C, respectively. The same pattern was seen for the concentration of carotenoids, whereas both cold and heat stress caused an increase in the concentration of these pigments (Table 1).

Total soluble protein content varied both among the treatments and basil parts. In leaves (Table 1), under the 4 °C treatment, a higher content of soluble proteins was measured, but without a statistically significant difference compared to the control. In all other treatments, both under cold and heat stress, a lower content of soluble protein was measured compared to the control. In roots (Table 1), a lower content of soluble proteins was measured only under the 10 °C treatment, while under the 4 °C treatment, as well as under 30 °C and 40 °C treatments, a significantly higher content of soluble proteins was measured compared to the control.

### 2.2. Activity of Antioxidant Enzymes

**Figure 1:**
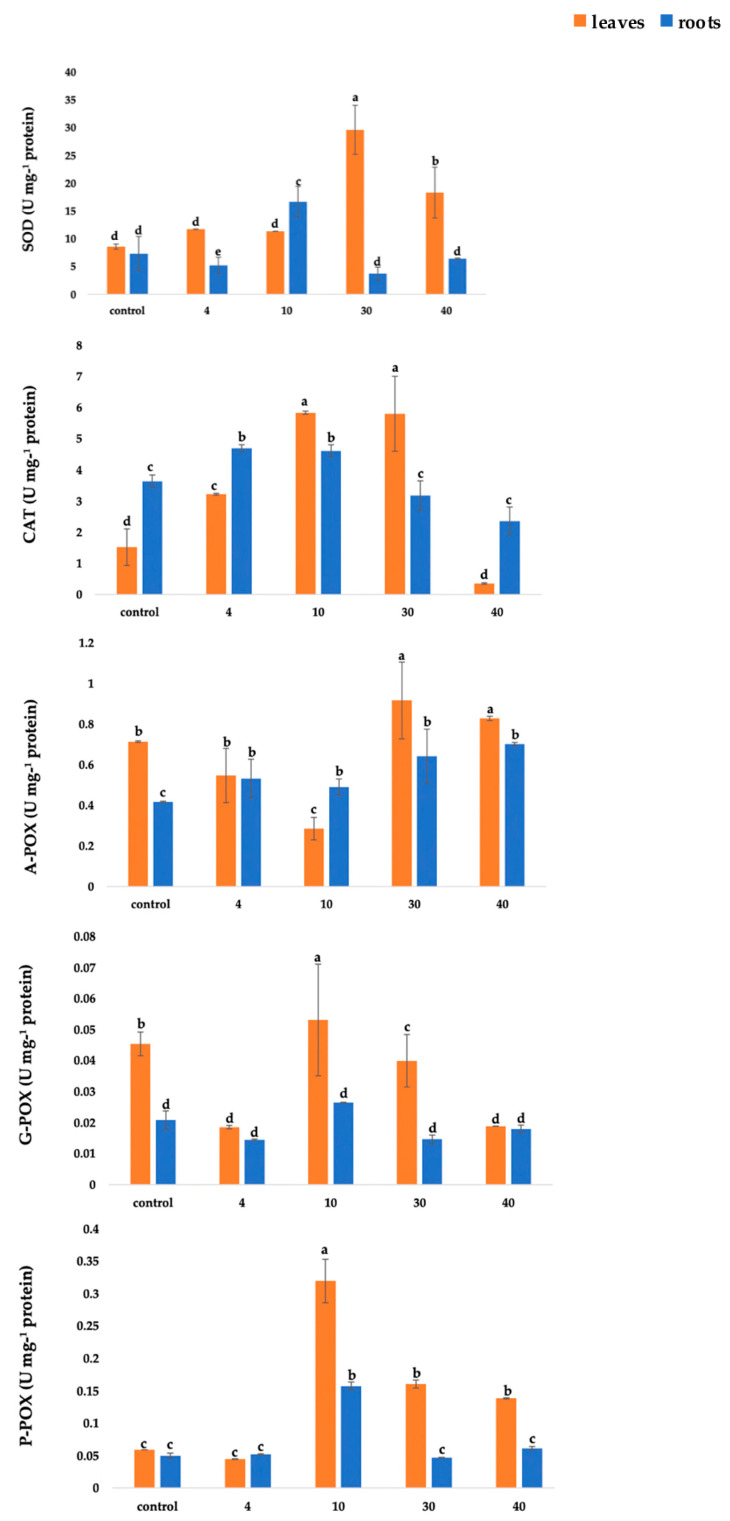
Activity of antioxidant enzymes in leaves and roots of *O. basilicum* under the conditions of temperature stress: 4 °C, 10 °C, 30 °C, and 40 °C. SOD—superoxide dismutase; CAT—catalase; A-POX—ascorbate peroxidase; G-POX—guaiacol peroxidase; P-POX—pyrogallol peroxidase. Different letters indicate significant differences (*p* < 0.05) among the treatments. Values are expressed as mean ± SE.

In basil leaves, the cold stress slightly increased the activity of superoxide dismutase (SOD) (Figure 1); however, the difference in activity was not statistically significant. On the contrary, heat stress (both 30 °C and 40 °C) caused several times higher activity of SOD, which was significantly different from both control and cold stress. Temperature stress also caused changes in SOD activity in roots (Figure 1). Significantly higher activity of SOD was measured at 10 °C, while under 4 °C, as well as under both heat-applied stresses, the activity of this enzyme was significantly lower. With the exception of under the 10 °C treatment, the activity of SOD was higher in leaves than in roots.

The activity of catalase CAT varied depending on the applied treatment and basil part (Figure 1). In leaves, both cold temperature treatments caused a significant increase in CAT activity compared to the control plants, and the highest activity was measured under 10 °C. Under the conditions of increased temperature, CAT activity was induced only under 30 °C, whereas under 40 °C, CAT activity decreased in basil leaves, which was significantly different comparing to the other temperature treatments but without a significant difference when compared to the control. A similar trend was seen in basil roots (Figure 1). The highest CAT activity was measured under cold stress treatments, whereas CAT activity decreased under the conditions of 40 °C, but the values were not significantly different compared to the control.

Ascorbate-peroxidase (A-POX) activity differed depending on the applied temperature treatment. In basil leaves, under cold stress, measured A-POX activity was lower compared to the control (Figure 1). In contrast, heat stress caused an increase in A-POX activity. For both heat treatments (30 °C and 40 °C), significantly higher A-POX activity was measured compared to the control, as well as compared to the cold treatments. Temperature stress also caused an increase in A-POX activity in basil roots (Figure 1), and activity was higher comparing to the control, but there was no statistically significant difference in the activity of this enzyme between treatments.

Guaiacol-peroxidase (G-POX) activity varied among investigated parts of basil (Figure 1). In leaves, and under cold temperature treatments, G-POX activity significantly increased under 10 °C; however, it was several times lower under 4 °C compared to the control, as well as to the treatment with 10 °C. Heat stress caused a decrease in G-POX activity, since the lower activity was measured for both heat-applied treatments. Cold temperature stress and heat temperature stress did not affect the activity of G-POX in basil roots, since there was no significant difference in activity of this enzyme comparing treatment with the control, as well as within the treatments.

Temperature stress caused changes in pyrogallol-peroxidase (P-POX) activity in leaves and roots of basil (Figure 1). The highest activity of this enzyme was measured under temperature treatment of 10 °C and in this treatment, activity was significantly different from control, and from treatment with 4 °C. Under the conditions of increased temperature (30 °C and 40 °C), activity of P-POX was induced and was significantly different compared to the control. However, there was no statistically significant difference among applied treatments. In basil roots, P-POX activity was induced only under 10 °C, whereas no significant difference was seen between other treatments, including control.

### 2.3. Content of Secondary Metabolites

The content of total phenolics was measured in leaves and in roots of basil, and the results are presented in Table 2. Total phenolic content in basil leaves varied significantly regarding the applied stress treatment, with the values several times higher compared to the control. The highest content of total phenolics was measured after 4 °C cold stress (227.29 mg GAE/10 mg DW), and the lowest content of total phenolics was measured under controlled conditions (2.35 mg GAE/10 mg DW). As is mentioned, heat stress also caused an increase in the content of total phenolics, but without a statistically significant difference among two treatments.

Temperature stress also caused a significant difference in total phenolic content in basil roots. The highest amount of total phenolics in basil roots was measured in treatment with 4 °C (761.41 mg GAE/10 mg DW). However, cold stress increased total phenolic content only in the case of 4 °C treatment, whereas a lower content of phenolics was measured at 10 °C compared to the control. Heat stress also caused an increase in total phenolic content, in particular 40 °C, since the content of total phenolics in this treatment differed significantly compared to the control. In all applied treatments, total phenolic content was higher in roots than in leaves.

Applied short-term temperature stress caused changes in flavonoid concentration both in leaves and in roots. The obtained values are presented in Table 2. In basil leaves, a higher concentration of flavonoids can be seen in all treatments compared to the control. The highest concentration was obtained in 10 °C treatment (27.51 mg QE/10 mg DW), followed by the concentration measured after 40 °C treatment (27.31 mg QE/10 mg DW). These concentrations differed significantly compared to the control in which the lowest flavonoid concentration was measured (19.69 mg QE/10 mg DW). Induced synthesis of flavonoids could also be seen in basil roots. The highest concentration of flavonoids was measured after 4 °C treatment (13.71 mg QE/10 mg DW), and the lowest after 10 °C and in the control (11.43 mg QE/10 mg DW and 11.96 mg QE/10 mg DW, respectively).

**Figure 2:**
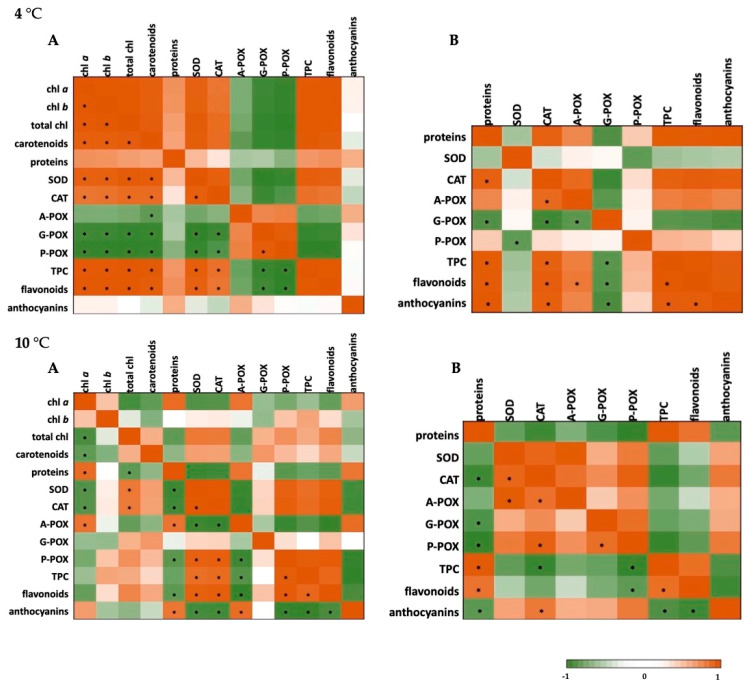
Heat map analysis of Pearson’s correlation for the targeted stress (4 °C or 10 °C) in leaves (**A**) and roots (**B**) of *O. basilicum*. Chl *a*—chlorophyll *a*; chl *a*—chlorophyll *b*; total chl—total chlorophyll; SOD—superoxide-dismutase; CAT—catalase; A-POX—ascorbate-peroxidase; G-POX—guaiacol-peroxidase; P-POX—pyrogallol-peroxidase; TPC—total phenolic content. * correlation is significant at *p* < 0.05; strong positive correlation (orange); strong negative correlation (green).

Total anthocyanin content in basil leaves (Table 2) was significantly higher after temperature stress. For all applied treatments, a statistically significant difference could be seen in the content of total anthocyanins compared to the control. The highest amount was measured after 30 °C treatment (17.69 mg cyn-3-gly/10 mg DW). The lowest content of anthocyanins was measured in leaves from control plants (8.35 mg cyn-3-gly/10 mg DW), with a significant difference compared to temperature stress treatments. The short-term cold temperature stress also increased the content of total anthocyanins in basil roots (Figure 2). The highest content was measured after 4 °C (10.02 mg cyn-3-gly/10 mg DW). However, after 40 °C treatment, a lower anthocyanin content was measured. This is also the treatment in which the lowest total anthocyanin content was measured (3.34 mg cyn-3-gly/10 mg DW).

### 2.4. Correlation Analysis and Heat Maps

**Figure 3:**
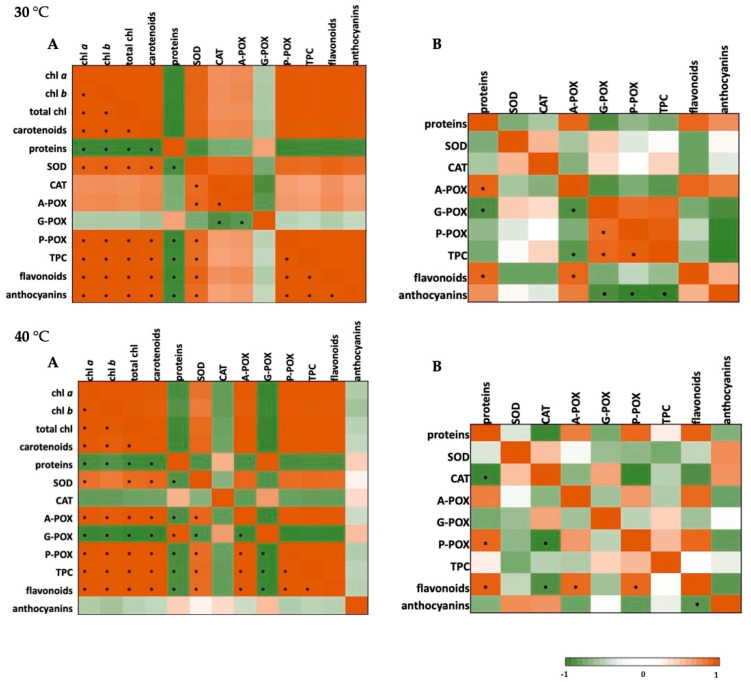
Heat map analysis of Pearson’s correlation for the targeted stress (30 °C or 40 °C) in leaves (**A**) and roots (**B**) of *O. basilicum*. Chl *a*—chlorophyll *a*; chl*a*—chlorophyll *b*; total chl—total chlorophyll; SOD—superoxide-dismutase; CAT—catalase; A-POX—ascorbate-peroxidase; G-POX—guaiacol-peroxidase; P-POX—pyrogallol-peroxidase; TPC—total phenolic content. * correlation is significant at *p* < 0.05; strong positive correlation (orange); strong negative correlation (green).

The data on photosynthetic pigments, soluble proteins, and both enzymatic and non-enzymatic components of the defense system for basil plants under the conditions of short-term cold and heat stress were analyzed using a Pearson’s correlation coefficient and presented using the heat maps. The results are presented in Figure 2 (cold stress) and Figure 3 (heat stress).

When it comes to the severe cold stress (4 °C), a significant positive correlation could be seen among photosynthetic pigments, SOD, CAT, phenolics, and flavonoids in basil leaves, as well as for CAT and A-POX, CAT and phenolics, flavonoids, and anthocyanins in roots. At 10 °C, in leaves, SOD and CAT were negatively correlated with A-POX and anthocyanins but positively correlated with P-POX, total phenolics, and flavonoids, while in roots, a strong positive correlation was found among SOD, CAT, A-POX, and phenolics and flavonoids.

When it comes to the heat stress at 30 °C (Figure 3), a significant positive correlation could be seen among photosynthetic pigments and antioxidant enzymes (SOD and P-POX), among SOD and both CAT and A-POX, as well as P-POX and secondary metabolites in basil leaves, whereas in roots it was seen for G-POX, P-POX and total phenolics. At 40 °C, in leaves, photosynthetic pigments were positively correlated with SOD, A-POX and P-POX, and a significant correlation can also be seen for secondary metabolites. In roots, severe heat stress caused a negative correlation of CAT and both P-POX and flavonoids, and a positive correlation of P-POX and A-POX with flavonoids.

## 3. Discussion

Climate change and increasing global food demand together with the negative impact of temperature change threaten the stability of food systems on national to global scales, causing a decline in yield of the major agricultural regions, crop production, and food security [15,16]. Temperature is among major environmental factors that are relevant to plant breeding since, beyond the temperature optimum, which is crop and variety specific, major yield loss occurs [17].

A decline in photosynthetic pigment content and reduced pigment synthesis is a common issue under stressful conditions due to reducing the uptake of Mg, improving chlorophyllase activity, damage of chloroplast apparatus, and generation of ROS. The decline in photosynthetic pigments has been shown under the conditions of low-temperature stress in the case of grapevine cultivars [7], tea leaves [8], tomato seedlings [18], and under the conditions of heat stress in wheat genotypes [19] and in cucumber seedlings [20]. Kalisz et al. [13] investigated the effects of prolonged chilling stress on several basil cultivars and concluded that chilling treatments did not decrease the extractable chlorophyll content in basil plants and showed genotypic differences and cultivar-specific alterations. According to Haldiman [21], the chilling-tolerant genotypes, when compared with the sensitive ones, displayed a higher content of chlorophyll and carotenoids at low temperature. In addition, an increased chlorophyll *a*/*b* ratio and decrease in chlorophyll to carotenoid ratio can be seen in heat-tolerant cultivar [5,22]. Al-Huqail et al. [14] showed total chlorophyll reduction in basil leaves under high temperature; however, the authors did not indicate a specific genotype used in this study. In our study, both short-term chilling and heat stress caused a significant increase in chlorophyll *a*, chlorophyll *b*, and carotenoid content in leaves of tested basil cultivar. Since it was demonstrated that genotype *O. basilicum* cv. ‘Genovese’ tends to decrease photosynthetic pigment content under unfavorable growth conditions [23], based on the results of this study it may be concluded that *O. basilicum* cv. ‘Genovese’ could tolerate short-term temperature stresses. Considering that temperature stress affects photosystem II (PSII) photochemistry and Fv/Fm ratio [6], and that the chloroplasts are the major sources of ROS, future studies should include additional photosynthetic parameters to support this hypothesis. This could be of particular importance because in recent years, the question has arisen whether improving antioxidant capacity followed with the reduction of chlorophyll and carotenoid degradation together with the lessening of PSI and PSII photoinhibition may help improve tolerance to temperature stress [24]

Abiotic stresses leads to changes in protein metabolism, and the synthesis of proteins take place as an important adaptive strategy in plants [25]. Proteins are important players in cold stress response and development after exposure to low-temperature stress, and the changes in protein levels may be critical for the creation of a cellular environment under low temperature [26]. Heat stress differentially affects the stability of various proteins, and plants respond to high temperatures by changing protein content [3,27]. In this study, the total soluble protein level in leaves of *O. basilicum* cv. ‘Genovese’ was higher under cold stress, and in roots under cold and heat stress, compared to the control plants. Under temperature stress, protein content is likely to differ in roots from shoots due to distinct molecular mechanisms of adaptation to environmental stresses, different stress tolerance, and temperature sensitivity [25]. Soluble proteins can be regarded as osmoprotectants (osmolytes) whose accumulation and increased content, as one of the protection mechanisms under stressful conditions, aid plants to maintain optimal balance and to preserve redox homeostasis and optimal plant metabolism [28,29].

Plant redox changes results in induction of various biochemical processes through regulatory network of antioxidants since under unfavorable temperature conditions increased H_2_O_2_ content can be observed, leading to lipid peroxidation in membranes and accumulation of malondialdehyde (MDA). Increased oxidative damage, measured as increased lipid peroxidation and H_2_O_2_ concentration, is a common response of genotypes that are non-tolerant to temperature changes [9]. Plants protect cells and subcellular systems against cytotoxic effects of the active oxygen radicals through the activity of antioxidant enzymes, and an antioxidant defense mechanism is activated to cope with both high and low-temperature stress in plants. However, overall antioxidant capacity differs between the species, and between tolerant and sensitive genotypes [30]. It is proved that, when it comes to basil, the activity of antioxidant enzymes under stress conditions depends on various factors including plant part, stress level, as well as tested cultivar [31,32]. Furthermore, in genotype *O. basilicum* cv. ‘Genovese’ coordination in an antioxidant defense system under stressful conditions protect membranes from lipid peroxidation and leads to a decrease in the MDA level [12]. Membrane damage followed by increased MDA level and decreased SOD, CAT, and peroxidase activities are biochemical responses in heat-sensitive genotypes, and there is a direct relation between temperature tolerance and increased activities of antioxidant enzymes, in particular SOD, CAT, and A-POX [9,30]. The first antioxidant enzymatic pathway in heat-tolerant genotypes utilizes SOD, CAT, and/or peroxidases located subcellularly in mitochondria, chloroplasts, and peroxisomes, since it was found that the activity of these enzymes increased with heat stress [33]. Among enzymes included in protection, SOD is often regarded as the first line of defense, and through the SOD activity, O_2_^−^ radical is converted into H_2_O_2_ and O_2_. CAT decomposes H_2_O_2_ without a cellular reducing equivalent; A-POX (class I peroxidase) catalyzes the conversion of H_2_O_2_ into H_2_O using ascorbate, while G-POX and P-POX (class III peroxidase) decompose H_2_O_2_ through oxidation of phenolic co-substrates (guaiacol and pyrogallol). In the current study, induced activity was recorded for SOD, CAT, A-POX, and P-POX in leaves under heat stress, which supports the notion that induction and regulation of SOD, CAT, and peroxidases are necessary for decreased membrane damage and substantial tolerance against heat stress [9,34]. Heat stability of different enzymes is essential for plant growth and survival under high temperature conditions, and according to Khanna-Chopra et al. [35], SOD is the most heat stable enzyme followed by A-POX and CAT. Authors demonstrated that SOD plays an important role in thermotolerance, since SOD protein is known to have a high melting temperature which may be one reason for its thermostability. Furthermore, according to authors, in young leaves of model system plant *Chenopodium album* increased SOD activity was followed by loss in leaf soluble protein content, whereas for a single CAT isozyme optimal temperature and stability is required, considering that the activity was showed only at up to 40 °C. This is in agreement with our results for young basil leaves under high temperature stress, which caused loss in leaf soluble proteins, induced SOD and A-POX activity for both heat-stress temperatures, and induced CAT activity only for 30 °C, while for 40 °C the activity of CAT decreased. To the best of our knowledge, there are no reports of antioxidant enzyme activity in basil under the heat stress conditions; however, our results are in accordance with previous research where heat stress caused the higher activity of SOD in ‘Carrizo’ citrange [36], and CAT and peroxidases in wheat genotypes [37] under 40 and 30 °C, respectively. Furthermore, as proved by statistical analysis, under the conditions of temperature stress significant positive correlation was found between various antioxidant enzymes and chlorophyll content, which is in accordance with the results of Almeselmani et al. [19].

Each cellular compartment contains more than one enzymatic activity that scavenges a particular ROS, and how these activities are coordinated between different compartments and within each compartment during temperature stress is an important question [38]. Whereas a significant protective role of SOD, A-POX and CAT was seen under heat stress, for leaves exposed to low temperature stress, the main enzymes included in protection were CAT, G-POX and P-POX. Plant responses regarding the cold acclimation are related to changes in cell wall composition and the activities of cell wall modifying enzymes [39]. The first target of a low-temperature stressor is the plasma membrane since oxidative stress causes lipid peroxidation and changes in membrane permeability [40]. As a result, lignification can occur during low-temperature stress as a prevention of damage and cell collapse [39]. Class III peroxidases, located both subcellularly and in cell walls and apoplast, play an important function in polymerization of cell wall components and cell wall modification and it may be presumed that under the chilling conditions in leaves of *O. basilicum* cv. ‘Genovese’, these enzymes protect cells through the ROS scavenging and cell wall modification. The induced activity of SOD, CAT, and peroxidase, and reduced A-POX activity under cold stress (10 °C) is in accordance with previous research of *Capsella bursa-pastoris* [41]. Our results are also in accordance with the report of Rezaie et al. [42], who investigated the response of antioxidant defense system in leaves of *O. basilicum* var. keshkeni luvelou after cold stress (4 °C and 10 °C) and recorded increased SOD, CAT, and G-POX activity; however, A-POX activity was induced, which is in contrast to our results for this enzyme. Kalisz et al. [31] reported a negligible alteration in CAT activity in ‘Genovese’ basil under cold stress. As was stated by Kalisz et al. [31] and confirmed by Rezaie et al. [42], such differences in activities of enzymes with similar function might be caused by cellular location, an imbalance among antioxidant enzymes, sensitivity to low temperature, and the phase of the plant’s response to the stressor.

Plants that are capable of removing reactive oxygen species efficiently are less sensitive to environmental changes, since the integrative biochemical and physiological responses of various components of the antioxidant defense system are essential in the avoidance of damage caused by oxidative stress. Plant secondary metabolites play a major role in overcoming stress conditions and in the adaptation of plants to the environment [43]. Although some metabolic changes are common to particular stresses while others are specific, a great number of metabolites changed in response to temperature stress testifying towards a strong impact of temperature on plant metabolism [44]. Under stressful conditions, under heat as well as under cold, plants synthesize more phenolic compounds, including phenolic acids, flavonoids, flavonols, and anthocyanins, which protect plant cells [45]. Induced synthesis of phenolics is accompanied by their assimilation into the cell wall as either lignin or suberin, since lignification and suberin deposition are shown to increase resistance to cold [43,46]. On the other hand, since the metabolism of phenolics takes place in the cytosol, it is believed that soluble phenolics are the scavenger of ROS under heat stress conditions [47]. Under the conditions of short-term temperature stress, young *O. basilicum* cv. ‘Genovese’ plants can produce a remarkably higher content of phenolic compounds, including flavonoids and anthocyanins, comparing to control plants, with the special emphasis on total phenolics and their content in roots, which is several times higher compared to leaves. A high content of phenolic compounds, particularly in roots of *O. basilicum* cv. ‘Genovese’ has been reported earlier for young plants grown under unfavorable conditions [12]. Higher total phenolic content and flavonoid concentration was reported in leaves of *O. basilicum* var. keshkeni luvelou after exposure to 4 °C and 10 °C [42], and the crucial role of soluble phenolics and anthocyanins in the protection of oxidative stress caused by heat was confirmed by Wahid [47]. Our results imply a significant protective role of phenolic compounds in young basil plants under the conditions of temperature stress, especially in the underground parts. Since secondary metabolites act as regulators of reactive oxygen signaling, the accumulation of secondary metabolites (such as phenylpropanoids and anthocyanins) increases antioxidant capabilities and reduces oxidative stress [48,49]. In general, plants tolerant to stress have higher levels of stress-related metabolites under normal conditions and also accumulate larger amounts of these metabolites under stressful conditions [44]. *O. basilicum* cv. ‘Genovese’ is a significant source of valuable metabolites whose synthesis can be induced under stress conditions [12] and our study showed that this genotype accumulates phenolic compounds under both heat and cold temperature stress. Under short-term heat stress conditions, thermostable SOD, CAT, A-POX, and P-POX in coordination with phenolics, flavonoids, and anthocyanins protect membranes and photosynthetic apparatus and a enable high content of photosynthetic pigments. Furthermore, an increased content of phenolics, flavonoids, and anthocyanins together with CAT, G-POX, P-POX increase under chilling stress, indicating the coordination of these metabolites in ROS scavenging and cell wall modification in protection against freezing damage. To progress understanding of multiple pathways involved in enzymatic and non-enzymatic defense against temperature stress, more experimental data are required including on ROS compartmental generation, overall accumulation, and removal through the investigated antioxidants.

## 4. Conclusions

Basil plants have wide areas of application and basil cultivation is important aspect of modern crop productivity. From a practical point of view, there have been only a few systematic studies on the temperature stress resistance of basil, which is surprisingly having in mind practical significance of such investigations on various cultivars of this important crop. Our study demonstrated that *O. basilicum* cv. ‘Genovese’ is capable of withstanding short-term temperature stresses. Furthermore, there are several merged responses for both cold and heat stresses in *O. basilicum* cv. ‘Genovese’, which are more related to severity than to the type of temperature stress. Both short-term cold and heat stress led to an increased concentration of photosynthetic pigments and osmolytes (soluble proteins). Although the activity of a particular enzyme varies under temperature stress, SOD, CAT, and various peroxidases can be induced in leaves of temperature-stressed plants and correlated with the content of photosynthetic pigments and secondary metabolites. While the activity of antioxidant enzymes was higher in leaves, roots of temperature stressed young plants showed a remarkable increase in secondary metabolite content (in particular total phenolics), testifying about the integrative physiological response of enzymatic and non-enzymatic components of the antioxidant system in the avoidance of damage. Results obtained in this study should contribute to a better understanding of temperature tolerance in *O. basilicum* cv. ‘Genovese’; however, further studies are required to attempt to elucidate the molecular basis of cold and heat tolerance. Moreover, additional photosynthetic analyses need to be included in future studies, since these data, together with the investigations on different cultivars with different duration and frequency of applied stress, will help to understand the dynamic phenotypes in various basil genotypes.

## 5. Materials and Methods

### 5.1. Experimental Design and Stress Conditions

Basil seeds (*Ocimum basilicum* L. cv. ‘Genovese’) were obtained from commercial source (SemeSemena D.O.O. Belgrade). The plants were raised in a growth medium that consisted of 7.57 mM NO_3_^−^, 4.77 mM K^+^, 1.37 mM Ca^2+^, 0.1 mM P, 0.5 mM Mg^2+^,0.07 mM Na^+^, 0.01 mM Fe^2+^, 7.38 μM Mn^2+^, 4.54 μM Zn^2+^, 1.91 μM J^+^, 4.33 μM B^3+^, 0.19 μM Mo, 0.11 μM Cu, and 0.12 μM Co [12] in vessels in environment cabinet (16/8 h light period, 60% humidity, temperature 23 ± 2 °C) and with one healthy seedling in each vessel. In all experiments, the similarly sized 4 weeks old plants were used with an average of five or six fully developed leaves. In order to apply temperature stress, controlled cabinet with stable temperature was used. The plants were divided into the following groups: the first group was the control group (25 °C). The second group was the chilled group (the 4 weeks old plants were subjected to cold stress and exposed to 4 °C or 10 °C for 4 h) according to Rooy et al. [7] with minor modifications. The third group was the heated group (the 4 weeks old plants were subjected to heat stress and exposed to 30 °C or 40 °C for 4 h). Three replicates were performed for each treatment, while one replicate comprised 30 seedlings. All the measurements were carried in triplicate.

### 5.2. Determination of Photosynthetic Pigments and Soluble Proteins Content

To ascertain concentration of photosynthetic pigments in leaves in both control and temperature stressed basil plants extraction was done in 80% acetone [50]. For each treatment, leaves from five individual plants for each of the replicates was used. Briefly, 0.5 g of fresh leaf material was homogenized in 10 mL of acetone, and then centrifuged at 700× *g* for 5 min. The content of photosynthetic pigments (chlorophyll *a* (Chl*_a_*), chlorophyll *b* (Chl*_b_*), total chlorophyll (Chl*_a_* + Chl*_b_*)), and carotenoids (C*_x_*_+*c*_) was estimated spectrophotometrically (Jenway UV/VIS 6105 with the resolution range 1 nm) and calculated using the following equations [51]:

Chl*_a_* = 12.21 × A_663_ − 2.81 × A_646_

Chl*_b_* = 20.13 × A_646_ − 5.03 × A_663_

Chl*_a_* + Chl*_b_* = 8.02 × A_663_ + 20.20 × A_646_

C*_x_*_+*c*_ = (1000 × A_470_ − 3.27 × Chl*_a_* − 104 × Chl*_b_*)/198.

The estimation of total soluble protein content in leaves and roots of basil was done with bovine serum albumin (BSA) as a standard [52].

### 5.3. Enzyme Extraction and Measurements

To determine the activity of antioxidant enzymes, extraction from leaves and roots was done using phosphate buffer with 1 mM EDTA, 2% polyvinylpyrrolidone (PVP) and 0.1% Triton X-100 [50]. For each treatment leaves and roots from five individual plants for each of the replicates was used. After the centrifugation at 12,000× *g* rpm for 20 min, the supernatant was collected and used to determine enzyme activity.

Superoxide-dismutase (SOD) activity was measured spectrophotometrically through the inhibition of nitro blue tetrazolium (NBT) photoreduction at 560 nm [53] and with the reaction mixture containing enzyme extract, 50 mM sodium-phosphate buffer (pH 7.8), 13 mM L-methionine, 10 mM EDTA, 1 mM NBT, and 0.2 mM riboflavin as the source of superoxide radicals. One SOD unit is the amount of enzyme that inhibits NBT reduction by 50% [53]. The SOD activity is expressed as U mg^−1^ of protein.

Catalase (CAT) activity is measured spectrophotometrically using method with ammonium molybdate [54]. The reaction mixture consisted of 65 mM H_2_O_2_, phosphate buffer, and enzyme extract was incubated at 37 °C for 1 min. By adding the 32.4 mM ammonium molybdate, the reaction was stopped and the complex of molybdate and H_2_O_2_ was measured against blank at 405 nm. One unit of CAT is the amount of enzyme that decomposes 1 μM of H_2_O_2_ per minute [54]; CAT activity is expressed as U mg^−1^ of protein.

To determine ascorbate-peroxidase (A-POX) activity, enzyme extract was added to the reaction mixture containing 50 mM phosphate buffer (pH 7), 0.5 mM ascorbate, and 0.1 mM H_2_O_2_ [55] and the production of dehydroascorbate was measured at 290 nm for 1 min. For calculation of A-POX activity, the molecular extinction coefficient ε = 2.8 mM^−1^ cm^−1^ was used, and enzyme activity is expressed as U mg^−1^ protein.

To determine guaiacol-peroxidase (G-POX) activity, enzyme extract was added to the reaction mixture containing 100 mM sodium-phosphate buffer (pH 7), 0.05% (*v/v*) H_2_O_2_, and 15 mM guaiacol. Oxidation of guaiacol to tetraguaiacol was observed based on an increase in absorbance at 470 nm during 1 min. G-POX activity was calculated using molecular extinction coefficient ε = 26.6 mM^−1^ cm^−1^ and expressed as U mg^−1^ protein. One unit of G-POX was defined as the amount of enzyme needed to produce 1 μM tetraguaiacol per min [55].

To determine pyrogallol-peroxidase (P-POX) activity, the enzyme extract was added to the reaction mixture containing 100 mM sodium-phosphate buffer (pH 7), 3.3 mM H_2_O_2_ and 20 mM pyrogallol. Purpurogallin was formed due to oxidation of pyrogallol and an increase in absorbance was observed at 420 nm during 1 min. Enzyme activity was calculated using the molecular extinction coefficient ε = 12 mM^−1^ cm^−1^ and expressed as U mg^−1^ protein [56].

### 5.4. Total Phenolic and Flavonoid Content

For quantification of total phenolic content and flavonoid amount in leaves and roots of basil plants, extraction was done using methanol and powdered air-dried plant material [57]. Collection of plant material was carried out by sampling leaves and roots from 10 plants of each treatment. For estimation of total phenolic content, 1 mg mL^−1^ of extracted material was added to the reaction mixture containing 10% Folin-Ciocalteu reagent and 7.5% NaHCO_3_. After incubation for 15 min at 45 °C, the absorbance was measured at 765 nm against blank (without extracted material). Total phenolic content was expressed in terms of gallic acid equivalent (mg GAE/10 mg DW). The flavonoid content was determined using a method with AlCl_3_, whereas absorbance was measured at 415 nm after 1 h of incubation. Results are expressed in terms of quercetin equivalent (mg QE/10 mg DW).

### 5.5. Total Anthocyanin Content

The quantification of total anthocyanin content was done using pH differential method which is based on structural changes in anthocyanin chemical forms [58]. The 1.5 mL of extract (obtained by sampling leaves and roots from 10 plants of each treatment) was mixed with 0.025 M potassium chloride buffer (pH = 1) and 1.5 mL of extract was mixed with 0.4 M sodium acetate buffer (pH = 4.5). The absorbance was measured at 520 nm and 700 nm for solutions pH = 1.0 and pH = 4.5. Total anthocyanin content was expressed as cyanidin-3-glucoside equivalent (cyd-3-glu) using the following equation:

$$\mathrm{TAC}\;(\mathrm{mg}/L)=\frac{A\times \mathrm{MW}\times \mathrm{DF}\times V\times 1000}{\varepsilon \times l\times W}$$

A—absorbance; MW—cyanidin-3-glucoside molecular weight (449.2 g mol^−1^); DF—dilution factor; V—solvent volume; ε—molecular extinction coefficient (26,900 L mol^−1^ cm^−1^), l—cell path length (1 cm). The results of total anthocyanin content are expressed in terms of cyanidin-3-glucoside equivalent (cyd-3-glu/10 mg DW).

### 5.6. Statistical Testing and Data Visualization

The significance of the differences among treatments was tested by one-way ANOVA at the 95% confidence level. The post hoc comparisons were done using the last significand difference test and the differences at *p* < 0.05 were considered significant. After normalization of the data, XLStat software, version 2020 (Addinsoft) was used to develop the heat maps after calculation of Pearson’s correlation coefficient.

## Figures and Tables

**Table 1:** The concentration of photosynthetic pigments (mg g^−1^ FW) and soluble proteins (mg g^−1^ FW) in *O. basilicum* cv. ‘Genovese’ after cold or heat stress.

Temperature Treatment (°C)	Chlorophyll *a*	Chlorophyll *b*	Total Chlorophyll	Carotenoids	Protein (Leaves)	Protein (Roots)
**control**	0.034 ± 0.003 c ^1^	0.024 ± 0.00 c	0.066 ± 0.007 b	0.016 ± 0.001 b	2.54 ± 0.03 a	3.52 ± 0.04 b
**4**	0.052 ± 0.000 b	0.037 ± 0.00 b	0.101 ± 0.002 a	0.024 ± 0.000 a	2.94 ± 0.09 a	4.84 ± 0.15 a
**10**	0.032 ± 0.007 c	0.023 ± 0.00 c	0.070 ± 0.017 b	0.017 ± 0.001 b	1.77 ± 0.02 b	2.06 ± 0.03 c
**30**	0.076 ± 0.001 a	0.047 ± 0.00 a	0.140 ± 0.001 a	0.030 ± 0.003 a	0.81 ± 0.01 c	4.30 ± 0.11 a
**40**	0.063 ± 0.001 a	0.032 ± 0.02 b	0.109 ± 0.021 a	0.030 ± 0.000 a	1.31 ± 0.01 b	4.34 ± 0.15 a

^1^ Different letters indicate significant differences (*p* < 0.05) among the treatments within each parameter. Values are expressed as mean ± SE.

**Table 2:** Total phenolic content (mg GAE/10 mg DW), flavonoid concentration (mg QE/10 mg DW), and total anthocyanin content (mg cyn-3-gly/10 mg DW) in *O. basilicum* after cold or heat stress.

Temperature Treatment (°C)	Total Phenolic Content		Flavonoids		Total Anthocyanin Content	
	Leaves	Roots	Leaves	Roots	Leaves	Roots
**control**	2.35 ± 0.07 d ^1^	452.00 ± 0.94 c	19.69 ± 0.01 d	11.96 ± 0.00 b	8.35 ± 0.00 d	4.18 ± 0.05 b
**4**	227.29 ± 0.52 a	761.41 ± 0.53 a	22.80 ± 0.00 c	13.71 ± 0.01 a	13.87 ± 0.02 b	10.02 ± 0.07 a
**10**	34.25 ± 0.53 c	314.35 ± 0.06 d	27.51 ± 0.01 a	11.43 ± 0.01 b	11.19 ± 0.08 c	5.51 ± 0.05 b
**30**	80.24 ± 0.35 b	449.65 ± 0.11 c	25.83 ± 0.01 b	12.19 ± 0.00 b	17.69 ± 0.03 a	4.68 ± 0.00 b
**40**	70.82 ± 0.12 b	470.82± 0.94 b	27.31 ± 0.00 a	12.43 ± 0.00 a	13.03 ± 0.17 b	3.34 ± 0.03 c

^1^ Different letters indicate significant differences (*p* < 0.05) among the treatments within each parameter. Values are expressed as mean ± SE.

## Data Availability

Not applicable.
